# Maintenance therapy for acute lymphoblastic leukemia: basic science and clinical translations

**DOI:** 10.1038/s41375-022-01591-4

**Published:** 2022-06-02

**Authors:** Linea N. Toksvang, Shawn H. R. Lee, Jun J. Yang, Kjeld Schmiegelow

**Affiliations:** 1 Department of Pediatrics and Adolescent Medicine, University Hospital Rigshospitalet, Copenhagen, Denmark; 2 Department of Pharmacy and Pharmaceutical Sciences, St Jude Children’s Research Hospital, Memphis, TN USA; 3 Khoo Teck Puat–National University Children’s Medical Institute, National University Hospital, National University Health System, Singapore, Singapore; 4 Department of Paediatrics, Yong Loo Lin School of Medicine, National University of Singapore, Singapore, Singapore; 5 Department of Oncology, St Jude Children’s Research Hospital, Memphis, TN USA; 6 Institute of Clinical Medicine, Faculty of Medicine, University of Copenhagen, Copenhagen, Denmark

**Keywords:** Acute lymphocytic leukaemia, Acute lymphocytic leukaemia, Chemotherapy

## Abstract

Maintenance therapy (MT) with oral methotrexate (MTX) and 6-mercaptopurine (6-MP) is essential for the cure of acute lymphoblastic leukemia (ALL). MTX and 6-MP interfere with nucleotide synthesis and salvage pathways. The primary cytotoxic mechanism involves the incorporation of thioguanine nucleotides (TGNs) into DNA (as DNA-TG), which may be enhanced by the inhibition of de novo purine synthesis by other MTX/6-MP metabolites. Co-medication during MT is common. Although Pneumocystis jirovecii prophylaxis appears safe, the benefit of glucocorticosteroid/vincristine pulses in improving survival and of allopurinol to moderate 6-MP pharmacokinetics remains uncertain. Numerous genetic polymorphisms influence the pharmacology, efficacy, and toxicity (mainly myelosuppression and hepatotoxicity) of MTX and thiopurines. Thiopurine S-methyltransferase (encoded by *TPMT*) decreases TGNs but increases methylated 6-MP metabolites (MeMPs); similarly, nudix hydrolase 15 (encoded by *NUDT15*) also decreases TGNs available for DNA incorporation. Loss-of-function variants in both genes are currently used to guide MT, but do not fully explain the inter-patient variability in thiopurine toxicity. Because of the large inter-individual variations in MTX/6-MP bioavailability and metabolism, dose adjustments are traditionally guided by the degree of myelosuppression, but this does not accurately reflect treatment intensity. DNA-TG is a common downstream metabolite of MTX/6-MP combination chemotherapy, and a higher level of DNA-TG has been associated with a lower relapse hazard, leading to the development of the Thiopurine Enhanced ALL Maintenance (TEAM) strategy—the addition of low-dose (2.5–12.5 mg/m^2^/day) 6-thioguanine to the 6-MP/MTX backbone—that is currently being tested in a randomized ALLTogether1 trial (EudraCT: 2018-001795-38). Mutations in the thiopurine and MTX metabolism pathways, and in the mismatch repair genes have been identified in early ALL relapses, providing valuable insights to assist the development of strategies to detect imminent relapse, to facilitate relapse salvage therapy, and even to bring about changes in frontline ALL therapy to mitigate this relapse risk.

## Introduction

Overall survival (OS) of acute lymphoblastic leukemia (ALL) has improved tremendously in recent decades and now exceeds 90% in children who receive the best contemporary therapy [1]. The path to this success was laid down more than half a century ago, when the folate analogue aminopterin (later replaced by methotrexate [MTX]) and the thio-substituted purine analogue 6-mercaptopurine (6-MP) were shown to induce temporary remission of ALL [2, 3]. Subsequently, remissions induced by vincristine (VCR) and glucocorticosteroids led to a steady increase in cure rates when remission was followed by maintenance therapy (MT) with oral daily 6-MP and weekly MTX until 2–2.5 years post remission [4].

In this review, we address the mode of action of MT, its necessary duration, strategies for dose adjustment and therapeutic drug monitoring, the impact of pharmacogenomic variants, mechanisms of relapse and drug resistance during MT, and novel approaches to improving MT.

## Maintenance duration

A meta-analysis of individual patient data from 3 115 children from 14 randomized trials investigating shorter vs. longer MT (2 years vs. 3 years or more) found that longer MT did not increase OS [5]. Further, there was no difference between boys and girls in terms of the effect of treatment length on event-free survival (EFS) and OS [5]. Male sex has historically been considered an adverse prognostic factor; consequently, on some protocols, male patients have received longer therapy than female patients. This sex-associated difference has, however, diminished with the advent of intensified, risk-based therapy, and although a recent study found that boys with B-cell ALL still experience inferior EFS and OS when compared to girls [6], longer MT for boys has largely been abandoned [4] (Supplementary Table 1). Reducing the total duration of chemotherapy to 18 months or less has been attempted, but this significantly increases the relapse rate [7]. Yet, even with truncation of chemotherapy at 1 year after diagnosis (6 months of MT), 60% of patients are cured [7]. However, identifying the subset of patients who need longer MT remains a challenge. Retrospective analysis of cytogenetic subsets indicated that more than 90% of patients with *t*(12;21)[*ETV6–RUNX1*] or *t*(1;19)[*TCF3–PBX1*] translocations were cured with only 1 year of chemotherapy [7]. There was no stratification of outcome analyses by the level of minimal residual disease (MRD) during the first months of therapy, and it therefore remains unclear whether MT can be shortened for patients who experience deep molecular remission after the first months of treatment.

## Methotrexate

**Fig. 1:**
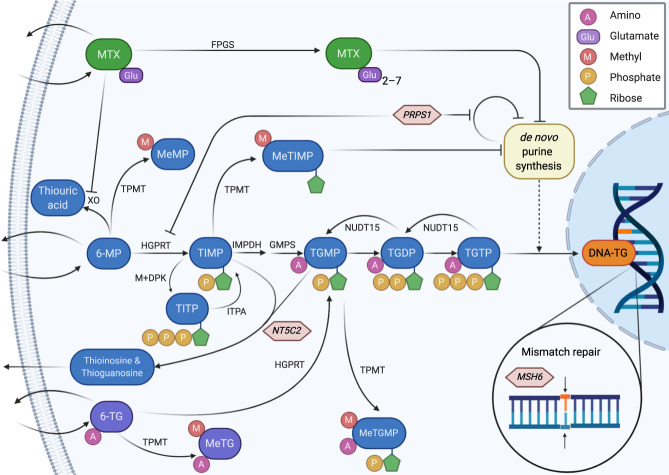
Thiopurine and methotrexate metabolism and mechanisms of thiopurine resistance. MTX is polyglutamated intracellularly by FPGS. 6-MP is metabolized through three competing pathways: conversion to thiouric acid by XO, methylation to MeMPs by TPMT, and conversion to TGNs. This multi-step process involves conversion to TIMP by HGPRT followed by conversion to TGMP by IMPDH and GMPS. Subsequently, deoxynucleoside kinases and reductase generate TGDP and then TGTP, which is incorporated into DNA (as DNA-TG) in competition with natural guanine. This process is counteracted by NUDT15, which dephosphorylates TGNs. Conversely, 6-thioguanine (6-TG) is converted directly to TGMP by HGPRT. Many of the intermediary thiopurine metabolites are substrates for TPMT, creating inactive metabolites (MeMP, MeTG, and MeTGMP), although MeTIMP is a potent inhibitor of de novo purine synthesis. Mutations in *NT5C2*, *MSH6*, and *PRPS1* illustrate mechanisms of thiopurine resistance resulting in early leukemic relapse. Figure created with BioRender.com. 6-MP 6-mercaptopurine, 6-TG 6-thioguanine, DNA-TG DNA-incorporated thioguanine, FPGS folylpolyglutamyl synthetase, GMPS guanine monophosphate synthetase, HGPRT hypoxanthine-guanine phosphoribosyltransferase, IMPDH inosine monophosphate dehydrogenase, ITPA inosine triphosphate pyrophosphatase, M + DPK monophosphate and diphosphate kinases, MeMP methyl-mercaptopurine, MeMPs methylated 6-mercaptopurine metabolites, MeTG methyl-thioguanine, MeTIMP methyl-thioinosine monophosphate, MSH6 MutS homolog 6, MTX methotrexate, NUDT15 nudix hydrolase 15, PRPS1 phosphoribosyl pyrophosphate synthetase 1, TGDP thioguanine diphosphate, TGMP thioguanine monophosphate, TGN thioguanine nucleotide, TGTP thioguanine triphosphate, TIMP thioinosine monophosphate, TITP thioinosine triphosphate, TPMT thiopurine S-methyltransferase, XO xanthine oxidase.

As an antifolate, MTX exerts its cytotoxicity by depleting reduced folates and directly inhibiting distal steps in nucleotide synthesis, thereby blocking thymidine and *de novo* purine synthesis (DNPS), which is paramount for the survival of leukemic stem cells [8, 9]. MTX is a pro-drug that is polyglutamated intracellularly by folylpolyglutamyl synthetase (FPGS), with up to seven gamma-linked glutamic acid residues (Fig. 1). Longer glutamate chains facilitate intracellular drug retention, as well as higher affinity for target enzymes in folate metabolism, such as dihydrofolate reductase (DHFR) [8]. Measurement of the cumulated MTXpg2–6 has been proposed as a means of therapeutic drug monitoring, eliminating the short-term fluctuation in MTXpg1 associated with MTX intake [10]. MTXpg4 dominates during MT, accounting for 30% of the long-chained MTXpg3–6 and having a 96% correlation with the variation in the summarized MTXpg3–6 [10].

The toxicity of MTX at low doses primarily manifests as moderate myelosuppression and hepatotoxicity, whereas high-dose MTX (HD-MTX), i.e., 24-h intravenous infusion of 5 g/m^2^ with subsequent leucovorin rescue, is associated with acute severe renal, neuro, and hepatotoxicity [11].

## 6-Mercaptopurine

The pharmacokinetics of oral 6-MP is characterized by low bioavailability, on average less than 20%, due to first-pass metabolism by xanthine oxidase in the intestinal mucosa and liver [12]. As a pro-drug, 6-MP undergoes extensive intracellular metabolism by enzymes in the *de novo* and salvage purine biosynthesis pathways, ultimately forming 6-thioguanine nucleotides (TGNs) [9, 13] (Fig. 1). These nucleotide analogs are then incorporated into the DNA double strand (as DNA-TG) in competition with natural guanine, with a median of approximately 1 in 6000 nucleotides being thioguanine (TG) substituted during MT [14]. DNA-TG can undergo random methylation, favoring mismatching with thymine (T). TG·T mismatching is recognized by the mismatch repair (MMR) system, with MutS homolog 6 (MSH6) playing a key role; however, as the aberrant base is in the template strand, it ultimately leads to DNA strand breaks and apoptosis [13]. Higher levels of DNA-TG have been associated with a reduced relapse hazard [14, 15]. There are many other intermediate thiopurine metabolites, some of which have also been linked to anti-leukemia effects. For example, thioinosine nucleotides and their methyl-derivatives (MeMPs) can directly inhibit DNPS [9, 13] (Fig. 1).

The complex processes by which thiopurines are metabolized give rise to wide inter-individual variability in the systemic exposure to these drugs. Consequently, both the efficacy and toxicity of thiopurines are highly variable, and a plethora of genetic factors (see below) and non-genetic factors have been implicated in influencing thiopurine pharmacology.

## 6-Thioguanine

Like 6-MP, 6-thioguanine (6-TG) exerts its cytotoxicity through DNA-TG, but its intracellular pathway is more direct, and early on it was regarded superior to 6-MP, e.g., higher potency and requiring a shorter duration of exposure for cytotoxicity [16]. 6-TG has been used mainly in the treatment of acute myeloid leukemia, but contemporary use also includes the consolidation phases of childhood ALL treatment, and long-term treatment of inflammatory bowel disease.

Three randomized trials have evaluated the replacement of 6-MP with 6-TG in childhood ALL MT, using 6-TG doses of 40–60 mg/m^2^/day [17–19]. In a meta-analysis of individual patient data from 4000 patients randomized in these trials, a significant benefit with respect to EFS was seen only in boys younger than 10 years of age (OR = 0.70; 95% confidence interval: 0.58–0.84); there was no benefit with respect to OS [20].

Patients receiving 6-TG exhibit seven-fold higher erythrocyte (Ery)-TGN concentrations when compared to patients receiving 6-MP [21]. However, when 6-MP is replaced with 6-TG, inhibition of DNPS by MeMPs is lost, which may account for the overall lack of improved efficacy in these trials. Consistent with this, the DNA-TG levels obtained with 6-TG and 6-MP administered in equipotent doses are almost identical [22]. Furthermore, patients receiving 6-TG experienced significant hepatotoxicity in the form of acute sinusoidal obstruction syndrome (SOS) (see below).

## Interaction of thiopurines and methotrexate

MTX increases the bioavailability of 6-MP by inhibiting xanthine oxidase, which catabolizes 6-MP [23] (Fig. 1). Inhibition of DNPS by MTX and MeMPs leads to increased levels of phosphoribosyl pyrophosphate, which can increase both the formation of TGNs and their incorporation into DNA [24]. There is a significant, albeit weak, correlation between Ery-TGNs and Ery-MTXpgs during MT [25]. DNA-TG is associated with Ery-TGNs, Ery-MeMPs, and Ery-MTXpg2–6 [26].

## Thiopurine Enhanced ALL Maintenance (TEAM) strategy

The addition of low-dose, slowly titrated 6-TG to the conventional MTX/6-MP maintenance backbone should, theoretically, increase DNA-TG markedly, because 6-TG leads to increased cytosol TGNs, and both MeMPs and MTXpgs will inhibit DNPS and, thus, enhance DNA-TG incorporation. In the recently piloted TEAM strategy, 2.5 mg/m^2^/day 6-TG is initially added to an MT backbone of 6-MP (50 mg/m^2^/day) and MTX (20 mg/m^2^/week) [27]. Subsequently, the 6-TG dose is increased in steps of 2.5 mg/m^2^/day at 2 weeks intervals to identify the maximum tolerated dose for the individual patient, up to a capping dose of 12.5 mg/m^2^/day. In this pilot study, 24 of 30 patients (80%) tolerated the maximum 6-TG dose [27]. When DNA-TG levels obtained with the TEAM strategy were compared with data from the Nordic Society for Pediatric Hematology and Oncology (NOPHO) ALL2008 trial, which included repetitive DNA-TG measurements in 918 patients with ALL [14], the TEAM strategy significantly increased DNA-TG levels (with a mean increase of 272 fmol/µg DNA; *P* < 0.0001). Such increments theoretically lead to a 59% reduction in the relapse hazard [27]. The TEAM strategy is now being tested in a randomized sub-protocol in the ALLTogether1 trial (EudraCT: 2018-001795-38).

## Adverse effects of thiopurines

Thiopurines are reasonably well tolerated, with myelosuppression being the most common dose-limiting toxicity. Pharmacogenetics strongly influence the risk of thiopurine-related myelosuppression, with variations in thiopurine S-methyltransferase (TPMT) and nudix hydrolase 15 (NUDT15) genes accounting for approximately 45% of the interpatient variability (see below).

Thiopurines frequently cause hepatotoxicity, mainly manifested as elevated serum aminotransferases without other signs of liver dysfunction [28]. The underlying pharmacologic mechanism is not clearly understood, although an association of transaminitis with high levels of MeMPs is established [29]. Fasting hypoglycemia during MT has also been associated with high levels of MeMPs [30]. Co-administration of allopurinol can reduce the level of MeMPs and alleviate hepatotoxicity and gastrointestinal toxicity through the inhibition of TPMT [31, 32]. However, this requires dose reduction of 6-MP, and the impact on relapse risk is unknown because TPMT low activity only moderately increases DNA-TG and a TPMT low activity genotype was not related to relapse risk in recent trials [33, 34]. Most importantly, allopurinol has not been tested in children with ALL in a randomized trial, although the combination of thiopurine and allopurinol has been shown to increase efficacy in patients with ulcerative colitis [35].

SOS is a severe hepatotoxicity, caused by disturbed microcirculation, that has mostly been reported with 6-TG therapy. SOS is one of the most frequent life-threatening complications of hematopoietic stem cell transplantation, with a mortality rate of 20% [36]. In contrast, SOS during chemotherapy is reported less frequently, can generally be managed conservatively or, in severe cases, with defibrotide, and is almost never fatal [37, 38]. In the three above mentioned randomized trials, 10%–25% of patients receiving 6-TG (40–60 mg/m^2^/day) experienced SOS [17, 19] or discordant thrombocytopenia [18] and 2.5% developed chronic hepatotoxicity including nodular regenerative hyperplasia (NRH) [39]. Even with short-term high-dose 6-TG during late intensification phases, the risk of SOS is increased [37]. Furthermore, the risk of developing SOS was 22 fold higher for *TPMT* heterozygous patients, as compared with *TPMT* wild-type patients (the general impact of TPMT is discussed below) [37]. Both the occurrence and severity of 6-TG-related hepatotoxicity appear to be highly dose-dependent, and it rarely occurs at doses below 12 mg/m^2^/day [39].

Both SOS and NRH are often accompanied by thrombocytopenia [20, 39]. Of note, high DNA-TG levels do not appear to be associated with an increased risk of SOS, nor with thrombocytopenia during MT [22]. In the TEAM pilot study, no hepatic serious adverse events (including SOS) were reported, and TEAM therapy was not associated with biochemical signs of increased hepatotoxicity or thrombocytopenia [27]. Therefore, the TEAM strategy is not anticipated to lead to excess hepatotoxicity in the form of SOS/NRH. However, with the introduction of new drugs for ALL such as inotuzumab, which can also cause SOS, especially if followed by hematopoietic stem cell transplantation [40], caution should be exercised when combining these agents, and further mechanistic studies are warranted to inform their proper use during antileukemic therapy.

## Measures of treatment intensity and novel biomarkers for therapeutic drug monitoring

Contemporary protocols use starting doses of 50–75 mg/m^2^/day for oral 6-MP, and 20–40 mg/m^2^/week for oral MTX (Supplementary Table 1). Doses are subsequently titrated to obtain a target degree of myelosuppression, as evaluated via the white blood cell count (WBC) or absolute neutrophil count (ANC), of which the ANC appear to be the most significant predictor of relapse [41]. However, there is no international consensus on dose titration strategies. Although some studies have associated dose intensity with EFS [42], this association has not been confirmed in more recent trials [14, 43]. Additionally, aggressive dosing may be counteracted by toxicities, and potentially increase the risk of developing a second malignant neoplasm (SMN) (see below) [13, 44].

Thrombocyte counts during and after the cessation of MT significantly correlate, but thrombocytopenia is rarely a dose-limiting factor [26]. Patients with unexplained thrombocytopenia should be evaluated for myelodysplasia, SOS/NRH, hypersplenism, and active viral infections (e.g., CMV or Parvovirus B19 infection).

Hepatotoxicity with high aminotransferase levels should not automatically lead to withholding of MT unless accompanied by bilirubin levels three times higher than the upper normal limit and/or coagulation factor II-VII-X levels below 0.50 IU/L [28], because patients who continue therapy have lower relapse rates than do patients with treatment interruptions due to hepatotoxicity [45]. High aminotransferase levels are a biomarker for patient adherence to MT, but recent studies have not found high aminotransferase levels to be associated with a reduced relapse rate [28]. Patients with liver dysfunction should be evaluated for causes other than MT, including hepatotropic viruses, SOS, or Gilbert syndrome. For patients with severe hypoglycemia, addition of allopurinol can be considered [32], although its impact on cure rates and DNA-TG levels is unexplored.

The traditional approach to guiding MT by monitoring the WBC/ANC is confounded by natural variation with age and ethnicity and by circadian and seasonal fluctuations [26]. Although Amerindian and African ancestries are established adverse risk factors in childhood ALL [1], the contribution of ethnicity-associated variations in normal WBC and ANC values is unknown. At the time of diagnosis of ALL, the normal level for each patient is unknown; therefore, applying a common WBC/ANC target for MT dose adjustment result in differing treatment intensities across patients. Hence, new strategies are needed to guide MT, and one based on DNA-TG may be a useful candidate, because (i) DNA-TG is a downstream metabolite that integrates upstream thiopurine and MTX metabolites; (ii) it is readily manipulable [27]; (iii) it has been linked to relapse, especially in patients who are MRD positive at the end of induction therapy [14, 15]; and (iv) monitoring of DNA-TG is feasible in multi-center studies, because it is very stable. Meanwhile, an optimal DNA-TG level that balance efficacy and toxicity has yet to be determined.

## Circadian schedule and co-administration of food

Historically, is was recommended to take 6-MP and MTX in the evening without concurrent food or milk intake [9]. However, two recent studies with a total of 973 children, and including 6-MP and MTX metabolite monitoring, found no association between the circadian schedule, metabolite levels, and relapse [46, 47]. Likewise, the supposed negative impact of food and milk intake, because of an anticipated effect of xanthine oxidase, was refuted [47]. Therefore, to promote treatment adherence, it is recommended that patients be instructed to follow a regular schedule without specific restrictions.

## Co-medication

Co-medication can skew the pharmacokinetics and pharmacodynamics of MTX and 6-MP. *Pneumocystis jirovecii* prophylaxis with trimethoprim-sulfamethoxazole enhances myelotoxicity and leads to lower administered doses of 6-MP, but it does not affect EFS [48]. Therefore, it is advised to administer this prophylaxis 2 or 3 days a week throughout MT to prevent potentially fatal *Pneumocystis* pneumonia [48].

Although widely used, VCR-containing pulses have been shown to prevent relapse in some trials, but with no clear effect on OS, whether combined with prednisone/prednisolone or with dexamethasone [5, 49] (Supplementary Table 1). However, they may be important in protocols in which less intensive treatment is given before MT [49] or in specific patient subsets such as those with *IKFZ1* deletion [50]. A recent Children’s Oncology Group (COG) trial showed no difference in OS when VCR/dexamethasone pulses were reduced from every 4 weeks to every 12 weeks for standard-risk patients [51]. The Chinese CCCG-ALL2015 trial also showed that removing VCR pulses after 1 year of ALL therapy did not compromise the cure rate for children with low-risk ALL [52]. VCR/dexamethasone pulses are currently being omitted in a randomization for patients stratified to intermediate risk-low treatment in the ALLTogether1 trial (EudraCT: 2018-001795-38).

CNS-directed therapy with intrathecal MTX (alone or combined with cytarabine and a glucocorticoid, i.e., triple intrathecal therapy) continues during all or part of MT, depending on the risk factors present [1, 4] (Supplementary Table 1). However, the spacing of intrathecal therapy makes it unlikely to cause noteworthy myelotoxicity and thus influence MTX/6-MP dosing. HD-MTX pulses with oral 6-MP are used in some protocols, although their benefit during MT has not been validated in randomized trials [11].

T-cell blasts have decreased sensitivity to many chemotherapeutics, including MTX, and many groups use HD-MTX or Capizzi-escalating MTX without leucovorin rescue during consolidation and/or MT for patients with T-ALL to enhance MTX efficacy [1, 11, 53]. Noteworthy, replacing MTX/6-MP MT with other chemotherapy seems to markedly increase relapse rate in T-ALL and high risk B-ALL [54]. A recent randomized study found an association of the purine nucleoside analog nelarabine with improved disease-free survival in patients with T-ALL. However, as other components differed, e.g., asparaginase dosing were more intensive in the nelarabine arm, the true impact of nelarabine remains uncertain. Regardless, several current protocols include nelarabine for patients with T-ALL, either for a selected subset of patients or for all patients with T-ALL [1, 53].

## Thiopurine pharmacogenomics

Genetic polymorphisms affect the competition between activation and inactivation metabolic pathways, thereby contributing to the interpatient variability in the efficacy and toxicity of thiopurine drugs, and these polymorphisms may be used to personalize treatment. The earliest example of the use of pharmacogenomics in ALL and the one most widely used clinically is *TPMT* genotyping. This is now a routine clinical test in many ALL consortia [55], and guidelines for individualized dose adjustment based on the TPMT genotype and/or phenotype are well established [56].

The TPMT enzyme methylates thiopurines and their intermediate metabolites, creating mainly inactive, but also some bioactive metabolites (MeMPs) [13, 56] (Fig. 1). TPMT activity shows monogenic, autosomal inheritance, and *TPMT* variant alleles that correlate with low enzymatic activity confer an increased risk of 6-MP toxicity through the accumulation of TGNs [33, 57, 58]. The frequency and type of variants affecting TPMT activity vary by ethnicity: 10% of Europeans have a genetic variant in *TPMT* and 0.5% are completely TPMT deficient, whereas TPMT deficiency is rare in East Asian populations. The *TPMT* gene is highly polymorphic, with a multitude of variants having been identified. Individuals carrying two loss-of-function *TPMT* alleles (homozygous or compound heterozygous *TPMT* deficient individuals) are at very high risk of life-threatening myelosuppression, if 6-MP dose is not appropriately reduced [56].

Historically, low TPMT activity has been linked to a reduced relapse rate concurrent with an increased risk of SMNs at standard 6-MP doses of 75 mg/m^2^ [59]. However, this effect disappeared in subsequent trials, that preemptively reduced 6-MP starting dose to 50 mg/m^2^ for *TPMT*-heterozygous patients [33, 34]. In accordance with these findings, *TPMT*-heterozygous patients receive the same 6-MP starting dose as do *TPMT* wild-type patients on the current European ALLTogether1 protocol (Supplementary Table 1).

Despite a lower frequency of *TPMT* mutations in Asians, they experience more thiopurine-induced toxicity compared to Europeans. A genome-wide association study (GWAS) revealed a variant in the *NUDT15* gene, predominantly found in patients of East Asian ancestry, that partly explained the ancestry-related differences in 6-MP tolerance [60]. *NUDT15* encodes a nucleoside diphosphatase that dephosphorylates TGNs, thereby preventing their incorporation into DNA [61] (Fig. 1), and one in 50 patients of East Asian ancestry shows an NUDT15 poor-metabolizer phenotype [56]. A recent study of 270 children enrolled in ALL trials in Guatemala, Singapore, and Japan identified three additional *NUDT15* variants associated with thiopurine toxicity [61].

The *NUDT15* genotype and activity are now comprehensively characterized, with massively parallel genotyping assays identifying almost 92% of all possible missense variants in *NUDT15*. These function-based variant classifications accurately predict risk alleles for thiopurine toxicity, vastly improving our ability to implement genotype-guided thiopurine therapy [62]. Similar to *TPMT*, *NUDT15* testing is now incorporated in clinical guidelines for thiopurine dose adjustment [56] (Supplementary Table 1), although the evidence supporting a different starting dose recommendation for patients who are intermediate metabolizers for both TPMT and NUDT15 remains limited [60].

The influence of the gene encoding inosine triphosphate pyrophosphatase (*ITPA*) has also been investigated. ITPA hydrolyzes thioinosine triphosphate (TITP) to thioinosine monophosphate (TIMP), thereby theoretically leading to increased levels of TGNs and DNA-TG; conversely, excessive TITP may be methylated by TPMT and contribute to the pool of MeMPs inhibiting DNPS [63] (Fig. 1). The evidence for the effect of ITPA remains conflicting. Inactivating polymorphisms in the *ITPA* gene have been associated with increased levels of DNA-TG [58]. Another study found that *ITPA*-heterozygous patients had significantly higher MeMPs levels compared to *ITPA* wild-type patients, which may lead to higher DNA-TG through increased inhibition of DNPS [64]. The presence of at least one nonfunctional *ITPA* allele has been associated with both improved and decreased EFS [65, 66]. Overall, prospective studies of this gene in larger multi-ethnic cohorts are indicated.

## MTX pharmacogenomics

Despite extensive studies of genes associated with MTX metabolism, there are currently no recommendations on MTX dosing based on genetic variants. Two single-nucleotide polymorphisms (SNPs) entailing reduced activity of methylene-tetrahydrofolate reductase (MTHFR), a key enzyme in the folate–homocysteine cycle, have been examined extensively in children with ALL. However, these studies collectively showed no evidence for any effects of these variants on MTX-related phenotypes [11, 55].

Furthermore, MTX pharmacogenomic studies have generally addressed high-dose rather than low-dose MTX; hence, the findings cannot be applied directly to dose adjustments during MT [11, 55]. In addition, MTXpg profiles have, thus far, not been associated with relapse risk [43].

One GWAS of 447 patients associated germline variants in *DHFR* and *FPGS* with short-chain MTXpgs and long-chain MTXpgs, respectively, and the variant in *FPGS* was also associated with increased relapse risk [67]. This implied that patients with the *FPGS* variant were sub-optimally treated, and thus, such patients may benefit from increased MTX doses relative to 6-MP doses. Interestingly, the *DHFR* genotype did not affect EFS in this study, possibly because short-chain MTXpgs are less potent than long-chain MTXpgs.

SNPs in solute carrier organic anion transporter family member 1B1 (encoded by *SLCO1B1*) have been found in GWAS to be associated with HD-MTX clearance [68]. Although not at genome-wide level, the same SNPs have also been implicated in MT. A study of 48 Turkish children found these variant alleles in *SLCO1B1* to be associated with lower MTX and 6-MP tolerance [69], and a separate study of 53 Japanese children found that polymorphisms in *SLCO1B1* was a predictor of 6-MP dose reduction [70].

## Polygenic risk scores

Given the complex metabolism of thiopurines and MTX, as well as their interplay, it is important to evaluate pharmacogenetic markers in a composite manner, determining the likely phenotypic effects of combination. Interactions among *TMPT*, *ITPA*, and *NUDT15* and their association with 6-MP toxicity have been described [60, 71]. However, large-scale studies to validate the utility of polygenic risk scores are lacking.

## Genomics of drug resistance and relapse

Besides affecting toxicity, genetic factors can also contribute to drug resistance via somatically acquired mutations. The current concept of leukemogenesis involves multiple subclones present at the time of diagnosis, some of which acquire additional mutations under the selection pressure of treatment, along with survival and expansion competition between subclones [72].

Gain-of-function mutations in cytosolic 5′-nucleotidase II (encoded by *NT5C2)* have been found to cause in vitro and in vivo thiopurine resistance [73–76]. The NT5C2 enzyme regulates the purine pool by dephosphorylating metabolites in the purine salvage pathway, but it can also dephosphorylate thiopurine monophosphate nucleotides (Fig. 1). *NT5C2* mutations are almost exclusively associated with early and on-therapy relapse, being present in 35%–45% of these cases [73–75], albeit often at a sub-clonal level. However, their presence is still associated with inferior outcomes in relapsed B-ALL [74, 76]. Moreover, in subsequent relapses, the *NT5C2*-mutated clones are often diminished or have even disappeared, which indicate impairment of their proliferative capacity [73, 76]. These observations suggest that *NT5C2*-mutated cells are not essential for the maintenance of relapsed leukemia, but they still play an important role in driving poor outcomes.

NT5C2 inhibitors are currently under development and investigation [77]. However, given the frequent sub-clonal nature of *NT5C2* mutations and their disappearance in subsequent relapses, it remains questionable whether therapy targeting the *NT5C2*-mutated cells at the time of relapse will be sufficiently effective [76]. Possible solutions to this problem, although yet to be tested, include targeting *NT5C2*-mutated cells during first-line therapy. Alternatively, early detection of *NT5C2* mutations may warrant treatment intensification with non–antimetabolite-based therapy [78].

Of note, germline *NT5C2* variants have been linked to both thiopurine metabolites during MT and with relapse-specific *NT5C2* mutations, indicating that there is an interaction between germline and acquired mutations, whereby primarily those patients with gain-of-function germline variants are more likely to develop relapse-specific *NT5C2* mutations [79].

The phosphoribosyl pyrophosphate synthetase 1 gene (*PRPS1*), which encodes the first rate-limiting purine biosynthesis enzyme, is also associated with early relapse [80]. Mechanistically, mutations in *PRPS1* lead to decreased feedback inhibition in the DNPS pathway, thereby increasing the pool of canonical purines competing with TGNs for incorporation into DNA. Furthermore, *PRPS1* mutations lead to decreased conversion of 6-MP to TIMP via competitive inhibition of hypoxanthine-guanine phosphoribosyl transferase by increased hypoxanthine levels [80] (Fig. 1).

Studies are underway to ameliorate drug resistance induced by *PRPS1* mutations. Inhibiting DNPS, either by CRISPR-Cas9 genome editing of de novo pathway genes or by treatment with lometrexol, a small-molecule inhibitor of DNPS that is in clinical development, can potentially reverse drug resistance [80]. *PRPS1*-mutant ALL cells have also been shown to be specifically more sensitive to 5‐fluorouracil (5‐FU) in both in vitro and mouse studies, highlighting 5‐FU as a potential chemotherapeutic agent for the salvage therapy of *PRPS1*-induced relapses [81].

Another mechanism of thiopurine resistance is malfunctioning of the MMR system, because the cytotoxicity of thiopurines is dependent on functional MMR (Fig. 1). Mutations in or copy number loss of *MSH6* have been found in 4%–10% of patients with relapsed B-ALL [82]. Knocking down *MSH6*, which is a critical component of the MMR system, not least for single-variant repair, leads to significant resistance to thiopurine therapy in vitro and in vivo. Hence, in these patients, despite their higher levels of DNA-TG, leukemic cells continue to proliferate [82].

Biallelic, constitutional deficiency in MMR systems usually causes increased mutability and, therefore, a hypermutator phenotype; however, reduced MSH6 activity in these leukemia clones has not been shown to result in an increased mutational burden or genomic instability [82]. Although treatment modalities to bypass *MSH6* mutations have yet to be identified, understanding the biological mechanisms of the mutations paves the way to ameliorating the resistance arising from these mutations. Immune checkpoint blockade is being investigated in MMR-mutated solid tumors (e.g., tumors of the colon and prostate and endometrial cancer); however, the efficacy of this treatment modality for ALL is in question in view of the putatively low mutational burden of ALL.

Recently, multiple relapse mutations have been identified in the *FPGS* gene, which encodes the enzyme that polyglutamates MTX, leading to MTX resistance [83].

Adding another level of complexity, epigenetic changes contribute to the clonal heterogeneity of ALL, but these changes are a not yet well understood. As an example, mutations in genes encoding the epigenetic regulators *CREBBP* and *WHSC1* have been found in relapsed ALL; however, the clinical significance of these findings, not least with respect to MT, remains to be determined [72, 74].

Most studies have failed to identify these relapse mutations in samples collected at diagnosis, suggesting that relapse mutations are acquired and promoted during treatment [73, 75, 77, 83]. However, one study found that 75% of relapsed B-ALL tumors were descendants of minor subclones already present at diagnosis [74]. A two-step process involving a pre-existing subclone that subsequently acquired additional mutations caused by chemotherapy and/or selection to proliferate has been proposed to be responsible for relapses emerging during MT [83]. Importantly, the new understanding of clonal evolution and the emergence of resistant subclones during therapy provide a strong rationale for the development and implementation of monitoring strategies to detect rising subclones, which have recently been piloted [83, 84].

## Carcinogenesis

Both the intensity of MT (evaluated by the average 6-MP dose) and its duration have been associated with the development of SMNs, most frequently myeloid neoplasms and CNS tumors [44, 85]. An association between the TPMT genotype/phenotype and SMNs has been shown in protocols using a 6-MP starting dose of 75 mg/m^2^/day [44, 85], but not in protocols using a 6-MP starting dose of 50 mg/m^2^/day [86]. The risk of developing an SMN appears to be highest in the standard-risk patient population, which could implicate their longer MT phase in some protocols, although the underlying factors, including mechanisms that drive both the propensity for *ETV6/RUNX1* mutation or high-hyperdiploidy [44] as well as SMN, have not yet been identified [87]. Although the cumulative incidences of SMNs in contemporary protocols are very low (around 1%–2%), it is crucial to prevent SMNs due to their dismal prognosis. This necessitates acquiring further understanding of the mechanisms underlying SMNs, and identification of patient subsets at high risk for SMNs through international collaborations, with extensive mapping of host genomic variants and characteristics of both ALL and SMNs.

## Maintenance therapy and quality of life

Even though MT is less intensive and toxic than the preceding treatment phases, it is long lasting, and its effect on quality of life (QOL) and how the treatment burden is perceived by patients and parents is still not fully elucidated. QOL during MT of children with ALL has been reported as significantly impaired, when compared to siblings or healthy children, and emotional reactions including fear, anger, sleeping problems, and worries, have been reported [88]. Studies investigating parents’ QOL during MT of their child have also reported sleep disturbances, high distress and low mental QOL [89]. When compared to parents with healthy children, the parents of children undergoing MT have higher scores for depression, but not for anxiety [90].

During MT, patients have less robust and stable sleep rhythms, lower levels of physical activity, and higher fatigue levels when compared to healthy children [91]. Moreover, patients experience even more fatigue and have lower physical activity when receiving dexamethasone-containing pulses, as compared to their experience during periods of MT without dexamethasone [91], emphasizing the importance of current and future studies aimed at de-escalating treatment intensity by skipping pulses, adding low-dose 6-TG to reduce 6-MP doses, or adding allopurinol to shift the thiopurine metabolism to a *TPMT*-heterozygous phenotype to decrease the burden of therapy while upholding survival outcomes [51].

## Treatment compliance and adherence

Treatment intensity during MT reflects both physician compliance with the treatment protocol and patient/parent adherence to therapy. Poor patient adherence has been reported in 10%–20% of pediatric patients with ALL. This varies with age and ethnicity and may be attributable to socioeconomic factors [92–94].

Poor adherence (defined as mean adherence rates < 90%–95%, as recorded by an electronic system registering bottle opening [95]) has been associated with a 2.5–3.9-fold increase in the risk of relapse [93, 94, 96, 97].

In addition to electronic monitoring, non-adherence may be revealed by low levels of drug metabolites with a rapid turnover, such as Ery-TGN/Ery-MeMPs/Ery-MTXpg [96, 98, 99], or alternatively, by an inability to reach the target myelosuppression level when doses are increased, not least when this is combined with no increase in serum aminotransferases as proxy measures [9].

Adherence is consistently reported to decrease in adolescents and young adults [92, 94, 100], and this may contribute to the inferior outcomes observed in these patients. Psycho-education and reminders have been attempted but have not been shown to improve adherence [97]. Hence, future studies should explore new strategies for monitoring adherence and interventions to mitigate this challenge. Three strategies for improving adherence are being tested in high-risk patients in the current COG AALL1732 trial (Clinicaltrials.gov identifier: NCT03959085).

## Conclusions and future directions

Over the past 60–70 years, ALL investigators have methodically tested different combinations of chemotherapeutics through successive clinical trials, and they have identified critical components of curative therapy for ALL. An important concept that emerged from these empirical efforts is the necessity of prolonged MTX/thiopurine MT.

From being a purely empiric, poorly understood, phase of anti-leukemic therapy, MT has recently become a focus of attention as a result of several basic science, genetic, and clinical studies. In the coming years, our understanding of MT and how to improve it will be facilitated by detailed monitoring of thiopurine and MTX metabolites combined with mapping of both host genetic variants and acquired mutations in relapse leukemia cells. Ultimately, this could lead not only to reduced relapse rates but also to the identification of patients who can be cured with less intensive and shorter MT. Should the TEAM strategy prove superior to conventional MTX/6-MP maintenance therapy without causing excess toxicity, future studies should investigate whether MTX can be omitted from a combined 6-TG/6-MP MT regimen, testing the hypothesis that DNPS can be inhibited sufficiently by MeMPs alone.

The prognostic impact of thiopurine and MTX metabolism has gradually diminished with the intensification of other drugs. For example, lower 6-MP dose intensity was significantly associated with higher incidence of ALL relapse in the St. Jude Total XIIIB trial, but it was no longer prognostic in more recent frontline ALL protocols evaluated by St. Jude and COG. This is also true for the effects of *TPMT* genotype on ALL treatment outcomes. Therefore, it is reasonable to speculate that MT could be de-intensified without compromising cure rates, at least for some patients (potentially those that are MRD negative at the end of induction therapy) yet identifying this subset of patients reliably remains a challenge. Interestingly, some ALL treatment protocols, especially those implemented in resource-limited countries, already feature less intensive MT (e.g., with lower thiopurine dosages to avoid infection), with which a significant proportion of patients are cured. Leveraging these “natural experiments,” one could retrospectively perform genomic profiling and identify features characteristic of patients who are cured in these settings.

The paradigm of ALL therapy is likely to shift significantly in the near future thanks to the introduction of several exciting novel therapeutics, e.g., blinatumomab, inotuzumab, and CAR-T cells. These immunotherapeutics have shown striking activity in relapsed and/or refractory ALL, and they are on track to move rapidly into frontline protocols. If these agents improve treatment outcomes for ALL, questions will naturally arise as to whether and which cytotoxic drugs should be eliminated from the protocols. In fact, a substantial proportion of patients with relapsed ALL who receive CAR-T therapy remain in remission even without MT. In the meantime, attention should be given to the impact of immunosuppressive drugs such as thiopurines and MTX on the efficacy of immunotherapy, if this is used in first-line therapy. Traditionally, it has been preferred to keep patients modestly myelosuppressed during ALL therapy, as this has been linked to a better prognosis. However, prolonged repression of host immunity may be detrimental to the activity of immunotherapeutics. Carefully designed clinical trials and correlative biology studies are urgently needed to determine the optimal timing and combination of various chemotherapeutic agents with immunotherapy in ALL treatment. As we inch toward a new era of ALL therapy, the field is wide open for the next generation of investigators to redefine MT by introducing more innovative, more precise, and less toxic regimens.

## Supplementary information

Supplementary table 1
