# Supplementary material for: Maintenance therapy for acute lymphoblastic leukemia: basic science and clinical translations
Source: Leukemia. 2022 Jun 2;36(7):1749–58. doi: 10.1038/s41375-022-01591-4 (PMC9252897; doi:10.1038/s41375-022-01591-4)
Supplement: Supplementary file 1 — Supplementary table 1 [file 41375_2022_1591_MOESM1_ESM.pdf]

Supplementary Table 1. Differences in maintenance therapy (MT) between current protocols.

Consortium	Protocol	6-MP starting dose *TPMT* and *NUDT15* wildtype (mg/m²/day) (oral)	6-MP starting dose<br><br>Heterozygous for *TPMT* and/or *NUDT15* low activity alleles (mg/m²/day) (oral)	6-MP starting dose<br><br>Homozygous for *TPMT* and/or *NUDT15* low activity alleles (mg/m²/day) (oral)	MTX starting dose<br><br>(mg/m²/week) (oral or intravenous)	Target ANC or WBC (x10^9/L)	Total ALL treatment duration	Pulses during MT	CNS prophylaxis during MT<br><br>(CNS1 only)
ALLIC[a]	ALLIC 2009	50	50, *TPMT* and *NUDT15* genetics not investigated on a regular basis	*TPMT* and *NUDT15* genetics not investigated on a regular basis	20 oral	WBC: 2.0–3.0<br>ANC: ≥0.2	104 weeks from start of induction	None	SR/IR B-ALL: IT MTX x 4<br>T-ALL and PPR: IT MTX x 6
AIEOP-BFM[a]	AIEOP-BFM ALL 2017	50	50	5	20 oral	WBC: 1.5–3.0	2 years from initial diagnosis	None	IT MTX/6 weeks x 6 in HR and patients with T-ALL
ALLTogether[a]	ALLTogether1	75	75	5	20 oral	ANC: 0.75–1.5	2 years from start of consolidation 1	SR: none<br>IR-low: randomization to omit VCR/dexa pulses/4 weeks<br>IR-high: VCR/dexa pulses/4 weeks throughout MT<br>HR: HD-MTX x 3	SR: IT MTX/12 weeks x 3<br>IR-low: IT MTX/12 weeks x 3<br>IR-high: IT MTX/12 weeks x 6<br>HR: IT MTX/6 weeks in MT1, /8 weeks in MT2
COG[1]	AALL0331 (SR B-ALL)	75	30%–50% reduction	10–20 mg/m²/3 days a week	20 oral	ANC: 0.5–1.5	2/3 years from start of IM1 in girls/boys	VCR/dexa pulses/4 weeks	IT MTX/12 weeks

COG[2]	AALL0232 (HR B-ALL)	75	30%–50% reduction	10–20 mg/m^2/3 days a week	20 oral	ANC: 0.5–1.5	2/3 years from start of IM1 in girls/boys	VCR/prednisone pulses/4 weeks	IT MTX x 1 or /12 weeks x 5 for patients who did/did not receive cranial irradiation
COG[3]	AALL0434 (T-ALL)	75	30%–50% reduction	10–20 mg/m^2/3 days a week	20 oral	ANC: 0.5–1.5	2/3 years from start of IM1 in girls/boys	VCR/prednisone pulses/4 weeks Randomized study: addition of 3 cycles of nelarabine for IR/HR patients	LR: IT MTX x 1 non-LR: IT MTX/12 weeks x 5
DFCI[a]	DFCI 16-001	50	50	N/A (adjusted based on tolerance)	30 intravenous	APC: 0.5–0.75	2 years from date of complete remission (end of Induction IA)	VCR/dexa pulses/3 weeks	B-ALL: TIT/ 9 weeks x 6, then /18 weeks<br>T-ALL: TIT/ 9 weeks
JCCG (JPLSG)[a]	JPLSG ALL B19	50 oral	50 oral	10 oral	20 oral	WBC: 2–3	Randomized 18–30 months from initial diagnosis[b]	LR: VCR/prednisolone pulses/4 weeks	LR: TIT/8 weeks x 5<br>SR: TIT/8 weeks x 2<br>IR: TIT/8 weeks x 4<br>HR: TIT/8 weeks x 6
Malaysia-Singapore[a]	Ma-Spore ALL 2020	50	37.5	5	20 oral	ANC: 1–2	15–18 months	SR/IR: VCR/dexa pulses/12 weeks<br>HR: VCR/dexa pulses/4 weeks	IT MTX/ 12 weeks in the first year
SJCRH[a]	Total Therapy 17	75	50–60	10	40 intravenous	WBC: 1.8–3.0	2.5 years from initial diagnosis	LR: VCR/dexa pulses/4 weeks for 1 year<br>SR: VCR/dexa pulses/4 weeks for 1–2 years	LR: TIT x 7 in the first year<br>SR: TIT x 10 in the first year

[a] Personal communication from Principal Investigators

[b] Females with HHD B-ALL randomized 24 months vs. 30 months. Females with other subtypes randomized 18 months vs. 24 months. Males with ETV6-RUNX1/TCF3-PBX1 randomized 18 months vs. 24 months. Males with other subtypes randomized 24 months vs. 30 months.

1. Maloney KW, Devidas M, Wang C, Mattano LA, Friedmann AM, Buckley P, et al. Outcome in Children With Standard-Risk B-Cell Acute Lymphoblastic Leukemia: Results of Children's Oncology Group Trial AALL0331. J Clin Oncol. 2020;38(6):602.

2. Larsen EC, Devidas M, Chen S, Salzer WL, Raetz EA, Loh ML, et al. Dexamethasone and High-Dose Methotrexate Improve Outcome for Children and Young Adults With High-Risk B-Acute Lymphoblastic Leukemia: A Report From Children's Oncology Group Study AALL0232. J Clin Oncol. 2016;34(20):2380–8.

3. Winter SS, Dunsmore KP, Devidas M, Wood BL, Esiashvili N, Chen Z, et al. Improved Survival for Children and Young Adults With T-Lineage Acute Lymphoblastic Leukemia: Results From the Children's Oncology Group AALL0434 Methotrexate Randomization. J Clin Oncol. 2018;36(29):2926–34.

6-MP, 6-mercaptopurine; AIEOP, Associazione Italiana di Ematologia e Oncologia Pediatrica; ALL, acute lymphoblastic leukemia; ALLIC; Acute lymphoblastic leukemia inter-continental; ANC, absolute neutrophil count; APC; absolute polymorph count; B-ALL; B-cell acute lymphoblastic leukemia; BFM: Berlin-Frankfurt-Münster; COG, Children's Oncology Group; DFCI, Dana-Farber Cancer Institute Consortium; dexa, dexamethasone; EORTC, European Organization for Research and Treatment of Cancer; HHD, high hyperdiploid; HR, high risk; IM, interim maintenance; IR, intermediate risk; IT, intrathecal; JCCG, Japan Children's Cancer Group; JPLSG; Japan Pediatric Leukemia/Lymphoma Study Group; LR, low-risk; MT, maintenance therapy; MTX, methotrexate; NUDT15, nudix hydrolase 15; PPR, prednisolone poor response; SJCRH, St Jude Children's Research Hospital; T-ALL; T-cell acute lymphoblastic leukemia; TIT, intrathecal methotrexate/cytarabine/hydrocortisone; TPMT, thiopurine S-methyltransferase; SR, standard risk; VCR, vincristine; WBC, white blood cell count.
